# Short-term outcomes after repair treatment (clipping or coiling) in aneurysmal subarachnoid hemorrhage (ASAH): a prospective multicenter study

**DOI:** 10.1186/2197-425X-3-S1-A781

**Published:** 2015-10-01

**Authors:** M Gero Escapa, D Iglesias Posadilla, J González Robledo, A Domínguez Berrot, A González Salamanca, L Nogales Martín, S Ossa Echeverri, A Diego Calvo, M Riesco Crespo, AM Olmos Linares, A Bueno Sacristán, R Ranedo Zaldo

**Affiliations:** Hospital Universitario de Burgos, Burgos, Spain; Complejo Asistencial Universitario de Salamanca, Salamanca, Spain; Complejo Asistencial Universitario de León, León, Spain; Hospital Universitario Río Hortega, Valladolid, Spain; Hospital Clínico Universitario de Valladolid, Valladolid, Spain

## Introduction

aSAH is a significant cause of morbidity and mortality, therefore early aneurysm repair treatment, surgical clipping or endovascular coiling, is mandatory to reduce the rate of rebleeding. Determination of treatment should be a multidisciplinary decision based on characteristics of the patient and the aneurysm, however there is variability among the different centers.

## Objectives

Analysis of outcomes and complications in patients with aSAH in regard to election of repair treatment modality and short term therapy.

## Methods

Multicenter, prospective and observational study. Including aSAH patients admitted in five neurocritical care units over 2014. Different variables were analized: morbidity (GOS at discharge), aneurysm location and size, repair treatment, aneurysm obliteration time, complications, ICU and hospital lenght of stay.

## Results

Sample size: 88 patients. Repair treatment: surgical(S)28,41%, endovascular (EVT) 56,82% and conservative 14,77%. Morbidity-GOS scale (S/EVT): 1 = 8 % /14 %; 2 = 4 %/ 2%; 3 =12 % /12 % 4: 28%/ 14% 5: 48 %/ 58% (p 0,59). We separated 2 GOS groups: good prognosis (grade 3,4,5) 88 %/84% and bad prognosis (1,2) 12 %/16% (p 0,64). Complications (S/EVT): vasospasm 28% /22% (p 0,57); rebleeding 24 %/10% (p 0,11) and delayed cerebral ischemia (DCI) 16%/34% (p 0,10). Aneurysm obliteration time (S/EVT): 3,92 days (SD 6,48) / 4,82 (SD 15,75) (p 0,79). Analysis of complications with short term therapy: < 72 h/ ≥ 72 h (72,97%/27,03%): vasospasm 24,07% /20% (p 0,711); rebleeding 9,26%/30% (p 0,026) and delayed cerebral ischemia (DCI) 25,93%/30% (p 0,726). Aneurysm location and treatment (S/EVT): anterior comunicant 5,48%/ 28,77%; posterior 9,59 %/ 12,33 %; middle cerebral artery 13,70%/ 13,70%, basilar 1,37%/2,74% and other 4,11%/8,22%. Aneurysmal sack diameter: small (< 15 mm) 27,78% /44,44%, large 4,17%/16,67% and giant (>25 mm) 1,39%/5,56%. Length of stay (S/EVT): ICU 26,32 (SD 4,54)/14,04 (SD 2,67) days (p 0,016) and hospital 36,8 (SD 6,04)/23,7 (SD 3,17) days (p 0,04).

## Conclusions

EVT grows up in the analyzed centers. In this study differences about outcomes and complications are not found in regard to repair treatment. In surgical patients rebleeding increases and in EVT DCI is greater. Short term therapy (< 72 h) decreases rebleeding. There is statistical significance in lenght of stay (ICU and hospital) decreases in EVT patients.

## References

[1] Sander E, Connolly (2012). Guidelines for the Management of Aneurysmal Subarachnoid Hemorrhage. Stroke.

[2] Lagares A (2015). Variability in the management of aneurysmal subarachnoid haemorrhage in Spain: Analysis of the prospective multicenter database from the Working Group on Neurovascular Diseases of the Spanish Society of Neurosurgery. Neurocirugía.

[3] Joanna D (2009). Long-Term recurrent Subarachnoid Hemorrhage after adequate coiling Versus clipping of ruptured intracranial aneurysms. Stroke.

